# Optimism bias regarding COVID-19: a cross-sectional study of lower-income older adults in Thailand

**DOI:** 10.1080/07853890.2023.2258893

**Published:** 2023-09-19

**Authors:** Paolo Miguel Manalang Vicerra

**Affiliations:** Shanghai University, Asian Demographic Research Institute, Shanghai, China

**Keywords:** Psychosocial behaviour, population ageing, social disparity

## Abstract

**Objective:** Understanding the discernment of individuals about their health is crucial during public health situations such as the COVID-19 pandemic. Within this theme of study is how older adults perceive their vulnerabilities because it can relate to subsequent disease preventing behaviour.

**Materials and methods:** The analysis explored optimism bias, or the perception of infection avoidance, regarding COVID-19 among lower-income Thais aged 60 and over. The study utilized an analytic sample of 2,139 individuals from the 2021 Survey on Housing and Support Services for Poor Older Adults. Logit regression model analysis was conducted, using optimistic bias as the outcome variable.

**Results:** Increasing age and residing in urban areas were associated with a higher likelihood of bias. On the other hand, higher educational attainment was found to decrease the association with optimistic bias, indicating higher perception of risks. Adherence of older individuals to the residence-in-place policy might have contributed to perception of lower infection risks. Urban residents had better access to welfare benefits and medical facilities, which led to reduced worry and greater optimistic bias.

**Conclusion:** Greater understanding of the disease and preventive strategies offer insights on how higher education levels lead to perceiving possible risks surrounding COVID-19.

## Introduction

The severe acute respiratory syndrome coronavirus 2 (SARS-CoV-2) is the cause of the coronavirus disease (COVID-19) [1]. It was suggested, even at the onset of the outbreak in 2020, that the virus was transmitted through the air, and certain social practices and environments contributed to its spread. Therefore, various measures were implemented across many societies to varying degrees of stringency, including sheltering in place, wearing face masks, and handwashing, among others [2]. Since COVID-19 was a novel disease, disseminating health information was also a crucial aspect of the pandemic response [3]. Building the capacity of societies to protect themselves against a disease that was not yet fully understood and had no direct treatment was challenging, as social and economic contexts varied, affecting individuals’ access to information and their willingness to comply with recommended health behaviours [4,5].

Understanding the breadth of individual and social psychology is crucial during unprecedented times like the current pandemic [6]. One particular theme that has been studied is the cognitive capacity of individuals and how they perceive their own vulnerabilities [7]. This pertains to people’s foresight and preparedness for future events. It is often the case that people emphasize the likelihood of positive events while underestimating negative outcomes [7]. This optimism bias has been explored in studies related to health [7,8]. Certain individuals with specific characteristics tend to exhibit this optimism bias regarding their health status, which influences their behaviour and their inclination to take risks. For example, a study of a campus influenza outbreak in the US found that some university students with unrealistic optimism had a lower intention to practice improved hygiene [9]. Another example involves older individuals, where those with an optimism bias had lower levels of knowledge about Alzheimer’s disease and less motivation to discuss it with medical professionals [10].

In the context of COVID-19 outbreak, health behaviour is influenced by social context [6]. Political polarisation, culture, and social norms can impact the effectiveness of behavioural change. Another significant factor associated with such change is social inequality [5,6]. While people may possess knowledge of the disease and public health recommendations, it may not always translate into practice. For example, purchasing face masks may be unrealistic for individuals who have lost their employment during the pandemic. In this regard, social heterogeneity has been found to be associated with optimistic bias [11–13]. Age, gender, educational attainment, socioeconomic status, and residence are some characteristics that have shown correlation with optimism bias. It is important to note that considering such bias needs to be explored in different societies due to policy and health contexts.

Thailand was among the first countries to detect a positive COVID-19 case in January 2020, which subsequently led to the implementation of a national lockdown policy by March 2020 [14]. After the first wave passed, most restrictions were lifted by June of that year, until the second wave of infections occurred around December 2020, lasting until about February 2021. Throughout the pandemic, it has been emphasized that certain population sectors, particularly older adults, are vulnerable [15]. Despite the repeated emphasis on precautionary public health messages, challenges in adherence were encountered among people in this age group, often associated with socioeconomic limitations [5,16].

The exploration of optimism bias, or unrealistic optimism, in the context of older adults in Thailand remains unexplored. This is a significant issue due to Thailand’s ageing population. This study focuses on lower-income older Thais and investigates the association between social factors, unrealistic bias, and susceptibility to COVID-19. The lifestyle and context of individuals in the lower-income category are often perceived as homogeneous, but it is crucial to comprehend their situation from a multidimensional perspective [17]. Understanding the social and economic disparities and their correlation with optimism bias can contribute to the enhancement of public health measures, extending beyond the current pandemic situation.

## Context and vulnerabilities of Thai older adults

The economic context of many older people in Thailand can be described as precarious, with 40% reported to be living below the poverty line in 2017 [18]. Filial piety remains an ideal, where children provide care and financial support to their older parents [19]. However, evidence suggests that income transfers between children and parents have been declining in recent decades [20]. Many older individuals continue to work, with approximately 40% reporting employment between 1994 and 2017, and around 35% indicating that their job is their primary source of income [21]. As many in this generation worked in the informal sector during their younger years, they are not members of any pension scheme. To address this, the government established a universal non-contributory pension scheme called the old-age allowance in 1990 [22]. Approximately 20% of the older population receive about US$ 20–33 per month through the scheme, but it remains insufficient to cover all the needs of this age group [23].

During the COVID-19 pandemic, the older population faced significant challenges due to mobility restrictions in their respective communities. This age group was identified as among the most vulnerable to the disease and, therefore, had to shelter in place [15]. However, many still needed to continue working to earn income [16]. Concerns about the unprecedented situation were widespread, as individuals were faced with the choice of protecting themselves from infection or earning a living. This had an impact on psychological distress and other health and well-being effects [24].

Other sources of risk and insecurity during the pandemic were already present in society before the events occurred. One such challenge was limited access to healthcare facilities for rural residents prior to the pandemic [25]. Although Thailand has a universal health coverage system in place, access can still be restricted due to geographic and logistical factors. Urban living, particularly in Bangkok, has also been noted to present challenges to well-being, including housing conditions for low-income individuals and built environments that are not conducive to those with physical limitations [16].

The aforementioned geographic and socioeconomic factors interact and contribute to disparities in the health and well-being of the older population. Similar factors have been recognized in the literature to influence the attitudes and behaviour of individuals during the pandemic in other societies [11,26]. One aspect that needs to be understood is how individuals themselves perceive their vulnerability. This understanding can aid in shaping health information programs that target people based on their social characteristics, as perceived susceptibility or invulnerability can influence individuals’ utilization of other medical services such as vaccinations and anticipatory care, among others.

## Methods

### Data and sample

In this study, the dataset analysed was the 2021 Survey on Housing and Support Services for Poor Older Adults. The respondents were individuals aged at least 55 years old from five regions of Thailand [27]. The socioeconomic criterion was based on individuals earning less than 40,000 Baht (∼US$ 1,330 at the time of writing) annually or those who were beneficiaries of the cash transfer program named ‘Card of the Poor’. The data was collected from May to June 2021, when national lockdown measures were relaxed in respective areas of the country, to determine the living conditions of the vulnerable sector. The primary aim was to gather general information, but COVID-19-related data was also collected for support purposes. A total of 2,139 respondents were recruited using a multistage cluster and stratification design for sampling. From the five geographic regions of Thailand, which are the North, Northeast, South, Central, and Bangkok regions, urban and rural communities were randomly selected. From the said communities, households that belonged to the category of ‘low-income’ described previously were selected.

For the analytic sample used in the current study, certain criteria were implemented. These criteria included: 1) excluding individuals who were bedridden, 2) cases where a proxy answered all the survey items on behalf of the older respondent, and 3) including individuals who were at least 60 years old to align with most studies concerning the older population. The resulting analytic sample consisted of 1,775 participants. Selectivity bias was tested due to the aforementioned exclusion criteria, and no statistical differences were observed in the distribution of basic demographic characteristics, such as age, sex, residence, living arrangement, employment, and education attainment, between the total sample and the analytic sample.

## Measures

### Optimistic bias

An item in the survey asked respondents about their perception of the risk of contracting COVID-19. The response options were ‘yes’ and ‘no’. Therefore, individuals who responded with the latter option were categorized as having an optimistic bias.

### Covariates

The covariates in the sample were grouped into sociodemographic characteristics, dimensions of poverty, and health status measures. These categories were based on previous studies that had observed their effects on the health attitudes and behaviours of older adults in Thailand [5,16,28]. Sociodemographic characteristics included age groups, gender, residence, and marital status. The region of residence was also included, with options of Bangkok, Central, North, Northeast, and the South. Living arrangements were categorized as living alone, living only with a spouse, living with children, living with grandchildren, or other arrangements, such as being a caretaker or living with siblings. Marital status has three categories: never married, previously married, and currently married.

Selected poverty dimensions focused on events within the scope of the pandemic period. These dimensions included employment status, income adequacy, skipping meals due to economic limitations, and perception of housing conditions. The perception of housing conditions is particularly relevant during the pandemic as households can be conducive to transmission when overcrowded [29]. The perception of housing conditions was created as an index based on four survey items asking respondents about issues related to their domicile, such as 1) small or overcrowded house, 2) distance from shops, 3) distance from health facilities, and 4) unsafe environment. A dichotomous variable was created to identify those with a poor perception of housing conditions, indicating at least two issues from the four items mentioned above.

Health status measures were all self-reported and included self-rated health during the past year and the presence of at least one chronic condition, such as neurological disease, osteoarthritis, chronic kidney disease, stroke, hypertension, diabetes, and hyperlipidaemia. Body mass index (BMI) was used in the analyses, calculated based on self-reported height and weight. Underweight was defined as BMI < 18.5 kg/m^2^; normal weight as BMI between 18.5 kg/m^2^ and 24.9 kg/m^2^; and overweight as BMI ≥25 kg/m^2^ [16]. Lastly, emotional difficulties were formed by combining various areas of emotional concern that respondents experienced at the time. Emotional distress here has been defined and measured in line with the literature [30,31]. These emotional concerns were: (1) being unable to have a health exam; (2) lacking a budget for medical treatments; and (3) being unable to commute to a medical facility. The reliability of the index constructed was estimated through Cronbach’s alpha with a value of 0.61, which was at the acceptable threshold of at least 0.6.

### Method of analysis

Characteristics distribution was conducted initially, followed by a bivariate analysis. The χ2 test was used to determine if there were statistical differences between non-optimistic and optimistic biases for each independent variable. Subsequently, the logit regression model analysis was employed with the optimistic bias as the outcome variable. Three models were utilized to examine the effects of different groups of covariates on the dependent variable [16]. Model 1 included only sociodemographic characteristics to assess disparities in gender and residence, among others. Model 2 explored the multidimensionality of poverty, while Model 3 examined disparities in health based on selected measures. The Wald test was performed for each model to assess the appropriateness of the included variables. Finally, the goodness of fit statistic was assessed for each model using the Akaike Information Criterion (AIC).

## Results

The analytic sample comprised a larger proportion of individuals in the young-old age group, specifically aged 60 to 69. This group predominantly consisted of females residing in Bangkok and urban areas. Moreover, the majority were not currently married and lived with their grandchildren (Table 1). Additionally, a significant number of participants had attained a primary level of education and were unemployed. Concerning measures of poverty, a considerable portion experienced insufficient income, despite being food secure and having a positive perception of their housing conditions. Furthermore, a higher percentage of respondents reported having poor self-rated health, normal BMI, and at least one chronic disease.

**Table 1.** Total sample distribution by optimism bias among lower-income older adults.

	Total (N)	Non-optimistic bias (%)	Optimistic bias	p-value
**Age**				
60-69	855	68.07	31.93	0.001
70-79	612	63.07	36.93	
80 and over	308	56.49	43.51	
**Sex**				
Male	632	66.46	33.54	0.166
Female	1143	53.17	46.83	
**Region**				
Bangkok	443	51.24	48.76	<0.001
Central	300	65.33	34.67	
North	404	75	25	
Northeast	410	64.88	35.12	
South	218	68.81	31.19	
**Residence**				
Rural	717	69.6	30.4	<0.001
Urban	1058	60.78	39.22	
**Marital status**				
Never married	126	54.76	45.24	0.011
Previously married	826	62.83	37.17	
Currently married	823	67.31	32.69	
**Living arrangement**				
Living alone	224	58.04	41.96	0.02
With spouse only	299	70.57	29.43	
Children	399	67.17	32.83	
Grandchildren	791	62.58	37.42	
Other	62	61.29	38.71	
**Education attainment**				
Lower than primary	289	58.13	41.87	0.046
Primary	1285	65.84	34.16	
Above primary level	201	63.68	36.32	
**Employment status**				
Employed	558	67.56	32.44	0.055
Unemployed	1217	62.86	37.14	
**Income inadequacy**				
Adequate	545	61.65	38.35	0.116
Inadequate	1230	65.53	34.47	
**Food insecurity**				
Did not	1496	64.1	35.9	0.634
Skipped meal	279	65.59	34.41	
**Perception of housing condition**				
Good	1583	63.11	36.89	0.002
Needs improvement	192	74.48	25.52	
**Self-rated health during past year**				
Poor	1086	66.21	33.79	0.039
Good	689	61.39	38.61	
**BMI level**				
Underweight	169	56.21	43.79	0.068
Normal	997	65.3	34.7	
Overweight	609	65.02	34.98	
**At least 1 chronic disease**				
No	596	64.43	35.57	0.954
Yes	1179	64.29	35.71	
**Emotional distress**				
No	1209	57.15	42.85	<0.001
Yes	566	79.68	20.32	
**Total**	1775	64.3	35.7	

Note: SRH: Self-rated health; BMI: Body mass index.

2021 Survey on Housing and Support Services for Poor Older Adults.

Bivariate analysis is also presented in Table 1, highlighting statistically significant differences (*p* value of <.05) in terms of optimism bias related to age, regional and urban-rural residence, and marital status, among other sociodemographic factors. Among individuals in the oldest age group, approximately 44% exhibited optimism bias, compared to 32% of those in their 60s. When comparing regions, nearly half of the residents in Bangkok displayed optimism bias, while around 39.2% of urban residents showed such bias, in contrast to approximately 30% of older people in rural areas.

Regarding educational attainment, individuals with lower than primary education exhibited the highest degree of optimism bias, at around 42%. Similarly, having a positive perception of one’s housing conditions was associated with a bias towards optimism, with approximately 37% displaying such bias. In terms of health status measures, self-rated health showed a correlation, where individuals with good self-rated health tended to be biased towards optimism. On the other hand, the experience of emotional distress was correlated with not having optimism bias.

Table 2 presents the results of regression analyses. The additive model analyses demonstrate that the inclusion of poverty measures and health status measures improved the observation of optimism bias, as evidenced by the decreasing value of the AIC and the increasing values of the Wald statistics. Notably, the statistically significant factors remained consistent between Models 1 through 3, even after including other groups of covariates. The same can be said for the poverty measures when comparing Model 2 and Model 3.

**Table 2.** Odds ratio of optimism bias by the covariates.

	Model 1		Model 2		Model 3	
	OR	(95% CI)	OR	(95% CI)	OR	(95% CI)
**Age** **(Reference: 60-69 years)**						
70-79	1.25*	(1.00-1.57)	1.25*	(0.99-1.57)	1.19*	(0.94-1.51)
80 and over	1.71***	(1.29-2.27)	1.63**	(1.21-2.19)	1.66**	(1.22-2.26)
**Female**	1.02	(0.82-1.28)	0.99	(0.79-1.25)	0.96	(0.76-1.22)
**Region** **(Ref.: Bangkok)**						
Central	0.57**	(0.41-0.81)	0.62**	(0.43-0.88)	0.74*	(0.51-1.06)
North	0.36***	(0.26-0.50)	0.38***	(0.27-0.53)	0.39***	(0.28-0.55)
Northeast	0.58***	(0.43-0.79)	0.65**	(0.48-0.90)	0.73**	(0.53-1.01)
South	0.51***	(0.35-0.73)	0.51***	(0.35-0.74)	0.57**	(0.39-0.85)
**Urban Residence**	1.10**	(0.87-1.39)	1.14*	(0.90-1.45)	1.14*	(0.89-1.45)
**Marital status (**Ref. Never married)						
Previously married	0.84	(0.55-1.28)	0.85	(0.55-1.29)	0.86	(0.56-1.29)
Currently married	0.86	(0.54-1.37)	0.87	(0.55-1.39)	0.88	(0.55-1.42)
**Living arrangement (Ref.: Living alone)**						
With spouse only	0.65**	(0.42-1.01)	0.63**	(0.40-0.98)	0.54**	(0.34-0.86)
Children	0.70*	(0.48-1.00)	0.69**	(0.48-0.99)	0.63**	(0.43-0.92)
Grandchildren	0.77	(0.56-1.07)	0.77	(0.55-1.07)	0.78	(0.56-1.08)
Other	0.78	(0.43-1.41)	0.77	(0.42-1.40)	0.78	(0.42-1.41)
**Education attainment** **(Ref.: Lower than primary)**						
Primary			0.70**	(0.53-0.93)	0.70**	(0.52-0.93)
Above primary level			0.8**	(0.53-1.20)	0.79*	(0.47-1.09)
**Unemployed**			0.98	(0.77-1.24)	1.00	(0.78-1.27)
**Inadequate income**			0.96	(0.77-1.21)	1.04	(0.82-1.32)
**Skipped meal**			1.00	(0.75-1.33)	1.16	(0.86-1.55)
**Needs improvement in housing condition**			0.64**	(0.45-0.91)	0.67**	(0.46-0.97)
**Good SRH in past year**					1.24*	(1.00-1.53)
**BMI level (Ref.: Normal weight)**						
Underweight					1.46**	(1.03-2.07)
Overweight					0.99	(0.79-1.24)
**At least 1 chronic disease**					0.94	(0.75-1.18)
**Emotional distress**					0.35***	(0.27-0.45)
**AIC**	1.275		1.274		1.235	
**Wald test**	76.11***		86.72***		104.23***	

Note: OR: Odds ratio; CI: Confidence interval; SRH: Self-rated health; BMI: Body mass index; AIC: Akaike Information Criteria.

***p < 0.001, **p < 0.01, *p < 0.05.

Being in the older age groups and residing in urban areas were observed to increase the odds of exhibiting optimism bias. Conversely, the opposite trend was observed for the measures of residence. Older individuals residing in regions other than Bangkok had a lower likelihood of displaying optimistic bias. Similarly, living with a spouse and children was associated with decreased odds of exhibiting this outcome. Furthermore, higher levels of educational attainment were shown to decrease the likelihood of bias, with primary education attainment having an odds ratio (OR) of 0.70, while attainment beyond primary level had an OR of 0.79. Individuals perceiving a need for housing improvements also had lower odds, with an OR of 0.67. Experiencing emotional distress was also observed to have an indirect association with optimism bias where the OR was 0.35. On the other hand, having good self-rated health (SRH) and being underweight increased the odds of optimism bias, with respective odds ratios of 1.24 and 1.46.

## Discussion

In the current study, the perception of susceptibility to COVID-19 among Thai older individuals was found to be associated with various socioeconomic, poverty-related, and health factors. These factors include older age, residing in rural areas, higher educational attainment, and having a positive perception of one’s health status, among others. However, these individual characteristics do not consistently exhibit the same direction of association, as they can be related to either realistic or non-realistic optimism.

Belonging to older age groups was observed to be associated with a lesser degree of optimism bias regarding vulnerability to COVID-19. This finding aligns with a similar result reported among older adults in Sweden during the early stages of the pandemic [32]. Public health measures in Thailand, particularly the implementation of a national lockdown, emphasized the need for older individuals to adhere to physical distancing and, whenever possible, stay at home. Such actions may have effectively increased the perception of vulnerability among older people. Additionally, it was found that individuals aged 70 years and above practiced preventive behaviours more frequently than those aged 69 years and below [5].

In relation to the previous point, rural older adults were also observed to exhibit better adherence to protective behaviours against COVID-19 [5]. It is counterintuitive for urban older adults to perceive themselves as less susceptible to the prevailing disease, despite potentially living in close quarters and having greater vulnerabilities in terms of nutritional status [27,33]. Urban areas in Thailand, particularly Bangkok, differ significantly from other geopolitical regions in terms of living conditions [25,34]. It has been noted that urban residents have greater accessibility to healthcare services [25] and are more likely to receive welfare benefits [34]. This perception of a supportive network may influence people’s approach to the situation.

Furthermore, the observation suggests that individuals with higher levels of educational attainment display less optimism bias regarding susceptibility to COVID-19. Similar differentiations based on education levels have been noted in another study on health-related risk perception [16]. It was found that individuals with higher education levels expressed concerns about their health potentially worsening during the pandemic and anticipated limitations in accessing healthcare services. The caution exercised by these individuals may be beneficial, as better-educated people in Thailand exhibited greater knowledge and adherence to recommended self-protective measures during the pandemic [5].

As emotional distress has been observed here to be associated with less optimism bias, it produces an ambivalence in desirability. Caution was heightened among low-income people in the country because it exacerbated their problematic condition pre-pandemic [24,25]. Their access to healthcare was challenging, and shelter-in-place regulations led to a situation where such physical and resource access to health facilities and goods was further constrained. Although having such concerns about the situation negatively impacts their quality of life [31], it was due to this caution that older individuals were seen to be more perceptive of the possibility that they themselves could be infected with the virus.

Several caveats must be mentioned regarding this study. Firstly, due to the use of cross-sectional data, causation cannot be established. Health-related information, such as height and weight for BMI, relied on self-reporting with no utilisation of biomarker data. Additionally, the operationalization of variables was constrained by the available information in the survey data. It is important to note that variables like housing dissatisfaction and inadequate food consumption were measured differently; therefore, comparing the presented results with other studies requires attention. Another limitation of this study is related to the data. As the aim of the survey used here was to assess the needs of lower-income people, the sample was limited to this group of individuals only. Comparing individuals from different income levels was not possible. In lieu of the information in the survey, characteristics of the people surrounding the older individuals who can influence the latter’s perception and mind-set about the pandemic could not be established.

Despite the aforementioned limitations, understanding patterns of health behaviour during emergency situations remains crucial. Individuals with diverse social characteristics and backgrounds will think and act based on their perceptions of the prevailing conditions. Older adults, in particular, are more vulnerable during such circumstances, necessitating efficient government planning and response. The implementation of programs that can provide timely and easily comprehensible information to the public is of paramount importance. These programs and approaches should also take into account the varying backgrounds of older individuals.
